# Host-Derived Extracellular Vesicles in Blood and Tissue Human Protozoan Infections

**DOI:** 10.3390/microorganisms11092318

**Published:** 2023-09-14

**Authors:** Natalia Tiberti, Silvia Stefania Longoni, Valéry Combes, Chiara Piubelli

**Affiliations:** 1 Department of Infectious, Tropical Diseases and Microbiology, IRCCS Sacro Cuore Don Calabria Hospital, 37024 Negrar di Valpolicella, Italy; silvia.longoni@sacrocuore.it (S.S.L.); chiara.piubelli@sacrocuore.it (C.P.); 2 Microvesicles and Malaria Research Group, School of Life Sciences, Faculty of Science, University of Technology Sydney, Sydney, NSW 2007, Australia; valery.combes@uts.edu.au

**Keywords:** protozoan infections, extracellular vesicles, malaria, Chagas’ disease, human leishmaniasis, human African trypanosomiasis, toxoplasmosis, biomarkers, host–pathogen interaction

## Abstract

Blood and tissue protozoan infections are responsible for an enormous burden in tropical and subtropical regions, even though they can also affect people living in high-income countries, mainly as a consequence of migration and travel. These pathologies are responsible for heavy socio-economic issues in endemic countries, where the lack of proper therapeutic interventions and effective vaccine strategies is still hampering their control. Moreover, the pathophysiological mechanisms associated with the establishment, progression and outcome of these infectious diseases are yet to be fully described. Among all the players, extracellular vesicles (EVs) have raised significant interest during the last decades due to their capacity to modulate inter–parasite and host–parasite interactions. In the present manuscript, we will review the state of the art of circulating host-derived EVs in clinical samples or in experimental models of human blood and tissue protozoan diseases (i.e., malaria, leishmaniasis, Chagas disease, human African trypanosomiasis and toxoplasmosis) to gain novel insights into the mechanisms of pathology underlying these conditions and to identify novel potential diagnostic markers.

## 1. Introduction

Parasitic diseases, including protozoan and helminth infections, are responsible for an enormous burden in tropical and subtropical regions, even though they can also affect people living in high-income countries, mainly as a consequence of migration and travel. Blood and tissue protozoan infections include malaria, which alone is responsible for the vast majority of deaths worldwide, notably in children under the age of 5, toxoplasmosis and a few neglected tropical diseases (NTDs), i.e., leishmaniasis, Chagas disease and human African trypanosomiasis. These pathologies are often, if not always, associated with poverty and are responsible for heavy socio-economic issues in endemic countries, where the lack of proper therapeutic interventions and effective vaccine strategies is still hampering their control. However, their investigation using either clinical samples or experimental models contributes to understanding their pathogenesis and revealing novel and important aspects of host–pathogen interactions. Among all the players involved in the pathogenesis of these diseases, extracellular vesicles (EVs) have raised significant interest during the last decades due to their capacity to modulate inter–parasite and host–parasite interactions [1].

### 1.1. Extracellular Vesicles: Important Definitions

EVs are well recognised as an important and unique mechanism of intercellular communication involved in the maintenance of homeostasis but also occurring during pathological conditions, including infectious diseases [2]. The release of EVs is a ubiquitous process across cell types and organisms, spanning from prokaryotes to eukaryotes, including humans, plants, helminths, fungi and algae [3]. These sub-micron elements are involved in inter-cellular, inter-species and inter-kingdom communication. They can indeed transfer biological material, including proteins and nucleic acids, from a parent cell to a recipient cell, while they might also carry markers indicative of their cellular origin [4]. EVs represent a heterogeneous group of vesicles in terms of biogenesis and molecular composition. For a long time, they have been divided into three main categories based on their size and biogenesis: exosomes (EXOs), which are endocytic vesicles originating within the multi-vesicular body and having a diameter < 100 nm; microvesicles (MVs, also known as microparticles—MPs, or ectosomes), originating directly at the plasma membrane through a budding process and having a diameter of approximately 100–1000 nm and apoptotic bodies, larger vesicles with a diameter > 1000 nm and released during cell apoptosis [5]. The increasing interest in EVs, particularly in their content and molecular functions, has led to a massive boost in the number of publications dealing with this subject since the beginning of the 21st century. However, this has been accompanied by increasing confusion and doubts regarding their nomenclature and the experimental methods employed to demonstrate their nature [6]. Even though some biophysical properties can be exploited to differentiate between vesicle populations (like size, morphology or protein composition)—as it was undertaken in the past—some overlaps between the different categories exist, and it is now evident that most often it is not possible to clearly identify and isolate a unique sub-group of vesicles. Consequently, it was deemed necessary to standardise EV research. To tackle this problem in 2013, the International Society for Extracellular Vesicles (ISEV), which gathers expert scientists in the EVs field, proposed the first guidelines, the objective of which was to contribute to harmonising and standardising EVs research [7]. These guidelines were further updated in 2018 [8], giving important recommendations to researchers working in the EV field. According to ISEV, extracellular vesicles (abbreviated EVs) should be the preferred generic term. Due to the lack of specific markers able to unequivocally indicate vesicle biogenesis, ISEV suggests using alternative nomenclatures indicative of EVs size, the presence of specific molecular markers, or their cellular/experimental origin [8] when referring to sub-populations. 

For convenience, in this review manuscript we will use the general term EVs when referring to unspecified vesicle populations, thus comprising both small and medium-large vesicles, while the term microvesicles (MVs) will be used when referring specifically to medium-large EVs (i.e., >100 nm in diameter), also as a synonym of microparticles. The term EXOs will instead be used for papers dealing with small EVs (i.e., <100 nm in diameter) enriched either by ultra-centrifugation (i.e., >100,000× *g*) or through specific exosome-enrichment kits.

### 1.2. Generalities of EVs in Parasitic Diseases

During infections, EVs can be released by both parasites and host cells. In the case of intracellular parasites, EVs are also released by infected host cells, adding an additional layer of complexity to the picture. When considering host-derived EVs in infectious diseases, it is thus of primary importance to distinguish between EVs released by (i) infected cells and thus potentially bearing pathogen-derived molecules; (ii) host cells activated in response to the infection; and (iii) the pathogen itself. The biological role of the first group of EVs is still largely debated, as it is not yet clear whether they represent important mechanisms of antigen/Pathogen Associated Molecular Patterns (PAMPs) presentation—thus involved in infection control—or of immune escape promoting parasite survival, even though these two aspects are probably not mutually exclusive and rather concurrent [2]. For instance, in several conditions, EVs have been shown to stimulate T cells, either indirectly following their uptake by antigen-presenting cells (APC) or directly as they can carry processed antigens, MHC class I and II molecules, as well as co-stimulatory molecules [9,10,11,12,13].

The complex composition of both the surface and inner cargo of EVs is directly linked to their ability to mediate cell-cell, cell-pathogen or pathogen-pathogen communication. It is now clear that parasites use EVs as a communication tool; parasite-derived EVs can in fact deliver their cargo of nucleic acids, proteins and other bioactive small molecules to other parasites or host cells, modulating the host response to the infection and consequently influencing disease progression [14]. The state of the art of parasite-derived EV literature has already been extensively reviewed elsewhere [3,15,16,17,18,19,20,21,22,23,24]. In the present manuscript, we will focus on studies specifically investigating circulating host-derived EVs in clinical samples or in experimental models of human blood and tissue protozoan diseases (i.e., malaria, leishmaniasis, Chagas disease, human African trypanosomiasis and toxoplasmosis) to gain novel insights into the mechanisms of pathology underlying these conditions and to identify novel potential diagnostic markers. Parasite EVs released in the environment will only be covered marginally and only when instrumental for the investigation of the host response to the infection via vesiculation.

## 2. Malaria

**Figure 1:**
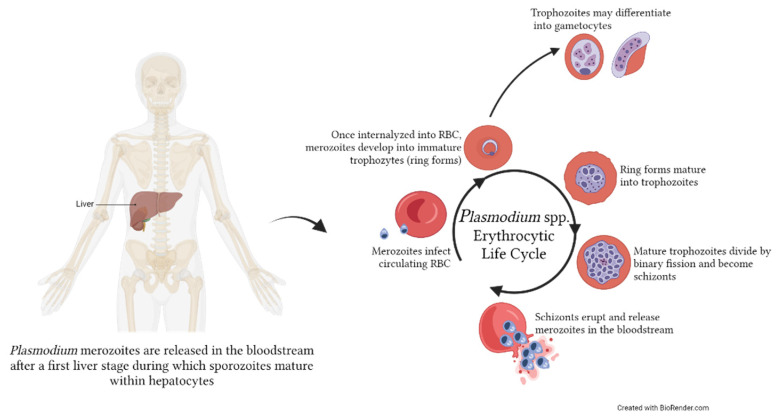
Erythrocytic life cycle of *Plasmodium* spp. within the human host. After a first initial liver stage, merozoites are released in the bloodstream, where they will infect red blood cells (RBC) to initiate the asexual intra-erythrocytic cycle. Within the RBC, merozoites become first immature trophozoites (ring stage), then mature trophozoites, and finally divide to become schizonts. These will eventually erupt from the RBC, releasing new merozoites in the bloodstream that will infect the new RBC. Occasionally, a portion of trophozoites will mature into gametocytes, which can be taken up by the mosquito vector. The image was created with BioRender.com.

Malaria is one of the most prevalent vector-borne parasitic diseases worldwide, affecting, in 2021, an estimated 247 million people and responsible for 619,000 deaths, mainly occurring in young children in sub-Saharan Africa [25]. The vast majority of malaria deaths are due to the development of complications (severe malaria—SM) of an otherwise uncomplicated infection. SM can manifest as different syndromes that can develop individually or in combination, the most common of which are cerebral malaria (CM), severe malaria anaemia (SMA), and respiratory distress (RD) [26,27,28]. Five different *Plasmodium* spp. can cause the disease in the human host, i.e., *P. falciparum*, *P. vivax*, *P. knowlesi*, *P. ovale* and *P. malariae*, among which the first three have been reported to cause severe malaria [29,30,31], even though most SM cases are due to *P. falciparum*. The malaria parasite has a complex life cycle, starting in the liver of the intermediate human host following the bite of the infected mosquito and then progressing to the blood circulation, infecting specifically red blood cells (RBC) at different stages of differentiation (e.g., reticulocytes for *P. vivax* and mature erythrocytes for *P. falciparum*) and finishing in the definitive host (mosquito) [32]. A graphical representation of the intra-erythrocytic life cycle of *Plasmodium* spp. is reported in Figure 1. 

From a pathophysiological point of view, SMA is associated with increased clearance of both parasitised RBC (pRBC) and non-infected RBC (nRBC) in the systemic vasculature, as well as altered haematopoiesis. CM pathogenesis is instead characterised by a combined uncontrolled inflammatory response and the mechanical obstruction of the deep microvasculature after the sequestration of pRBC and other host cells such as platelets and leukocytes, leading to microhaemorrhages and oedema in the tissues, notably in the brain, as well as the release of EVs following cellular activation [33,34]. 

In the context of malaria, little is known about the role of EVs in SMA pathogenesis, while host-derived EVs have been widely studied in CM using clinical samples, in vitro assays, and murine models (experimental CM). Even if the relevance of the murine model of CM is still debated, it has undoubtedly allowed the accumulation of valuable data and the testing of various hypotheses, which have later been confirmed in the clinical setting and have significantly contributed to our understanding of CM immunopathology [35,36,37]. 

### 2.1. Surface Markers of Circulating EVs during Malaria

As mentioned in the introduction, cells can release EVs in the extracellular space following activation, and, in some cases, these EVs will present on their surface markers specific to their parent cells as a result of the vesiculation process. The enumeration and surface phenotyping of EVs circulating in body fluids of infected subjects or laboratory animal models might thus serve as disease biomarkers, as they can be indicative of a specific pathology or stage of disease progression. The recent progress in flow cytometry and other detection techniques capable of reliable analyses in the sub-micron range has allowed collecting important information on the dynamic composition of circulating EV populations, revealing their potential as disease biomarkers. As described in the following paragraphs, alterations of specific EV subsets were observed in both malaria clinical samples and in the murine model (Table 1). 

#### 2.1.1. Clinical Malaria

A potential role for circulating MVs of endothelial origin bearing αv sub-unit/CD51, an endothelial marker released in deep tissues due to pRBC sequestration, was already hypothesised in 2004, when the number of circulating endothelial MVs (E-MVs) was found to be significantly increased in Malawian children suffering from malaria complicated with coma [38]. Additional studies corroborated this observation, showing the elevation of different cell-specific MV populations in the circulation of Cameroonian children with CM and further supporting the hypothesis that pan-cellular MV release is a feature of malaria complicated by coma. Indeed, children from Cameroon suffering from CM displayed increased circulating MVs of platelet, endothelial, erythrocytic, monocytic, and, to a lesser extent, lymphocytic origins when compared with children with SMA, uncomplicated malaria (UM), or controls [39]. In agreement with previous observations [38], the level of total, platelet (P-MV), endothelial (E-MV) and erythrocytic (R-MV) MVs significantly decreased at discharge, re-enforcing the association between severe infection and MV release. Interestingly, in CM patients, the number of circulating P-MVs negatively correlated with clinical parameters such as coma depth and duration, sustaining the potential involvement of immune cell-derived EVs, particularly those of platelet origin, in disease pathogenesis. P-MVs could be both the result of platelet loss and/or sequestration, and inversely correlated with platelet count, but could also represent important pathophysiological mediators of cytoadherence, as shown in vitro [47]. 

The release of MVs from pRBC (R-MVs) during malaria was later investigated in depth in clinical samples and in vitro in an effort to establish not only the level of circulating vesicles associated with different disease states but also the mechanisms or conditions underlying this release [40]. In particular, adult patients from Thailand displayed elevated circulating R-MVs compared to uninfected control subjects, irrespective of the infecting species (i.e., *P. falciparum*, *P. vivax* or *P. malariae*). However, *P. falciparum* severe malaria showed the highest levels of R-MVs, also correlating with peripheral parasitaemia [40]. 

Interestingly, in a large population from Ghana, increased circulating MVs were reported in patients compared to uninfected controls, independently of disease severity [41]. In this particular population, MV numbers were higher with younger age and showed a positive relation with parasitaemia in an age-dependent manner, indicating the potential of circulating MVs as diagnostic markers [41]. 

A multi-analyte panel targeting 40 protein features was employed to obtain a broader EV surface phenotyping in *P. falciparum* malaria patients [42]. Among those tested, CD106, CD81, HLA-DR and osteopontin were highlighted as significantly altered on EVs from malaria patients compared to EVs from healthy donors and were able to discriminate between the two groups with an area under the ROC curve (AUC) ranging from 0.92 to 0.97. This ability increased to 0.99 when those parameters were considered in combination with platelet count and heparin-binding EGF-like growth factor (HB-EGF). Those preliminary results indicate that the investigation of the surface proteome using both targeted and untargeted approaches might be an important tool to identify disease biomarkers and novel diagnostic targets and improve current rapid diagnostic testing [48,49]. 

The majority of EV studies have focused on *P. falciparum* malaria as the leading cause of death among malaria infections; however, increased circulating EVs released from platelets, RBCs and leukocytes were also detected in uncomplicated *P. vivax* patients from Brazil compared to controls, with P-EVs correlating with clinical manifestation [43], supporting the importance of these vesicular elements as markers of infection for other forms of malaria. Interestingly, plasma-EVs from *P. vivax* patients were reported to induce modulation in gene expression in human fibroblasts in vitro [44]. In particular, they induced a specific upregulation of ICAM-1 expression, a well-known marker of cytoadherence in malaria [50,51], suggesting a role for these EVs in mediating adhesion of patient-derived reticulocytes [44].

#### 2.1.2. Experimental Malaria

As previously mentioned, important findings have been collected in the murine model of CM, contributing to a better understanding of the role of EVs in malaria and, notably, CM pathogenesis [36]. The increased presence of P-MVs associated with thrombocytopenia was, in fact, first shown in the mouse [45]. Although at that time it was still difficult to discern between fragments derived from platelet degeneration and actual EVs, the study showed for the first time the inverse relation between platelet count and circulating P-MVs and proposed a role for caspases in this platelet loss and P-MV release [45]. Murine CM has also been essential in supporting the implication of host-derived EVs in disease pathogenesis. Indeed, mice in which cellular vesiculation in response to a vesiculation agonist was genetically impaired via the knockout of the ATP binding cassette A1 (ABCA1) gene, controlling externalisation of phosphatidylserine (a marker of some MVs), displayed resistance to CM, i.e., did not develop the neurological syndrome following infection with *P. berghei* ANKA strain (PbA) [52]. 

Murine CM was also particularly useful to closely follow the release of EVs during the course of disease progression, and the number of circulating P-MVs, E-MVs and R-MVs was shown to peak at the time of the onset of the neurological syndrome [46]. Importantly, MVs from PbA-infected mice also displayed a functional implication in disease pathogenesis since they were reported to sequester in the brain microvasculature when adoptively transferred to infected recipient mice. Conversely, MVs from stimulated endothelial cells transferred to uninfected mice triggered a CM-like pathology in both the brain and lungs (i.e., microhaemorrhages, perivascular oedema, and leucocyte sequestration), providing additional evidence for an active role of MVs in CM pathogenesis [46]. 

Even though some contrasting results in circulating EV enumeration have been reported, mostly due to the variability in both methodological aspects and characteristics of the studied population, it is clear that malaria infection triggers the release of MVs in the bloodstream. This process seems to be more prominent during severe disease, even though it also occurs in uncomplicated malaria. It remains to be evaluated whether this phenomenon is associated with poor immunity to malaria, since the data currently available seem to suggest that in areas of high endemicity, it affects mainly severe cases, while in areas of low/seasonal endemicity, it also occurs in uncomplicated infections.

### 2.2. Circulating EV Cargo: Phenotypic Characterisation and Functional Properties

When studying EVs, in addition to knowing their levels in the circulation of patients and their surface phenotype, it is paramount to characterise their inner cargo and understand their potential functional roles. Various approaches, such as high-throughput proteomics or next-generation sequencing, have been used to decipher the molecular components of this cargo, among which proteins and microRNAs (miRNAs) are the most investigated using clinical samples and experimental models (Table 2 and Table 3).

#### 2.2.1. MVs Protein Cargo

The proteomics investigation of the inner cargo of circulating MVs from malaria patients showed that malaria patients-MVs bore a significantly higher number of proteins compared to MVs released either from *P. falciparum* in vitro culture or from healthy subjects. Surprisingly, 1311 proteins were found to be present only in clinical malaria-MVs, some of which also displayed an association with peripheral parasitaemia. Proteins significantly associated with clinical malaria MVs included haemoglobin subunits and proteins involved in inflammation, coagulation and the complement pathway [53]. The increased cargo of haemoglobin (Hb) and complement-associated proteins suggests the involvement of circulating MVs in RBC clearance and Hb elimination during malaria, thus contributing to anaemia. Circulating MVs were also enriched with Rab proteins, some of which are considered master regulators of vesicular transport and trafficking [65]. All these pieces of evidence support the hypothesis that a specific protein packing into EVs might occur during malaria infection, and especially during CM, and that this cargo is important for the functional role of EVs in malaria pathophysiology and in promoting inflammation. Noteworthy, 29 P. falciparum proteins were also detected within circulating MVs, including Pf-enolase and merozoite surface proteins (MSPs). This observation supports the importance of circulating EVs as a source of diagnostic markers. 

The protein cargo of malaria-associated MVs was also investigated using the CM murine model [54]. The quantitative analysis of blood-derived MVs in susceptible mice infected with PbA revealed 60 proteins modulated in abundance, and 21 present only in acute CM. The cargo of circulating MVs was modulated during the course of the disease, and MVs released during the neurological phase were enriched with proteins involved in endothelial and immune cell activation. Additionally, the increased presence of Hb observed in clinical samples was also confirmed using the murine model. Two proteins, S100A8 and carbonic anhydrase I (CA-I), were further validated as associated with CM-derived MVs in different mouse strains and in a small number of clinical samples of children with CM and healthy controls. Although only two parasite-derived proteins, i.e., MSP-1 and intra-erythrocytic *P. berghei*-induced structures protein 1 (now exported protein IBIS1), were identified, the study proved the strong association between MVs protein cargo composition and the severe disease state [54].

#### 2.2.2. EXOs Protein Cargo

In the field of malaria, among the different sub-populations of EVs, MVs (i.e., medium/large EVs) are the ones that have predominantly been characterised in patients, in vitro and in vivo, while EXOs (i.e., small EVs) have not been explored as circulating markers of disease but rather as vehicles transferring their cargo to target cells.

The first report on circulating EXO protein content in malaria dates back to 2011, when Martin-Jaular and colleagues examined EXOs released during *P. yoelii* infection in mice [55], a rodent species that predominantly infects reticulocytes and, depending on the strain, can generate a non-lethal infection or a lethal one with the development of severe anaemia [37]. LC-MS/MS analyses showed that blood EXOs from infected mice mainly originated from reticulocytes, as they were enriched with reticulocyte proteins. Importantly, 31 parasite-derived proteins were detected, including MSPs, enolase and HSPs [55,66], as observed in MVs [53,54]. EXOs displayed immunomodulatory properties when used in immunised mice and elicited the production of parasite-specific antibodies, attenuated the course of the infection following challenge with a lethal *P. yoelii* strain [55] and triggered the production of splenic memory T cells [66]. 

Proteomic analyses of circulating EXOs showed the importance of EVs as a source of disease markers not only for *P. vivax* infection but particularly for hypnozoite infection. Indeed, using a liver-chimeric humanized mouse model for *P. vivax* infection, it was shown that circulating EXOs carry liver stage-specific biomarkers that include both host- and parasite-derived proteins [56]. Importantly, some parasite proteins were detected within circulating EVs only at specific time points post-infection. Similarly, plasma-EVs from *P. vivax* patients were reported to contain parasite proteins, including MSP-3, *plasmodium* exported protein (PHISTc) and GAPDH, even though their distribution was variable across samples [44]. When CD71^+^-circulating EVs were specifically enriched, the number of parasite-derived proteins and their distribution across samples increased, and 48 proteins were detected. Of these, MSP 3.1 and PHISTc showed antigenic properties since they were specifically recognised by patients’ serum [57]. Similarly to *P. falciparum* patient-derived EVs, in vesicles from *P. vivax* patients, Hb subunits were among the most abundant host proteins, together with acute phase proteins [57]. These observations suggest that specific proteins are packed within EVs during *P. vivax* malaria and that these EVs are important mediators of cytoadherence and immune response, supporting their potential as tools for diagnostic and vaccine antigen discovery [44,57].

Based on these pieces of evidence, it is clear that malaria infection is associated with alterations in the number, phenotype and molecular cargo of circulating EVs. By carrying parasite antigens and inflammatory molecules, they actively participate in the pathophysiological mechanisms. From a functional perspective, important aspects of host–pathogen and pathogen–pathogen interactions have been discerned through the in vitro study of EXOs released by pRBC. These include some elegant studies that are now considered important milestones. Indeed, pRBC-EXOs have been reported to promote parasite differentiation into gametocytes [67,68] and to mediate the transfer of drug resistance between parasites [68]. pRBC-EVs were also shown to display immunomodulatory properties in macrophages and neutrophils [67,69] and to modulate gene expression in endothelial cells via the transfer of miRNA [70]. Despite their doubtless relevance in the malaria-EVs filed, in vitro investigations of EVs released by pRBC are beyond the scope of this review manuscript and will not be described in detail.

#### 2.2.3. EXOs miRNA Cargo

Another important component of EVs inner cargo are small non-coding RNAs (sncRNAs), which act as master regulators of gene expression. EVs can deliver this genetic information to distant target cells, altering their gene expression and function [71,72]. MicroRNAs (miRNAs) represent the most studied sncRNAs associated with malaria EVs for their potential as disease biomarkers and their implication in disease pathogenesis. Indeed, as highlighted in Gupta et al., miRNAs are important immune regulators during malaria infection, and specific miRNAs are associated with severe complications or with different infecting *Plasmodium* species, representing potential targets for diagnostic and therapeutic interventions [73,74,75,76]. Interestingly, miRNAs packaged in EVs appear to be enriched up to 100-fold and are more stable and resistant to RNase digestion in body fluids than EV-free nucleic acids [77]. Specific miRNAs were quantified within circulating EXOs from patients (Thailand), and miR150-5p and miR51b-5p were highlighted as specifically associated with *P. vivax* infection, while let7a-5p was enriched within EVs from both *P. vivax* and *P. falciparum* compared to uninfected EVs [60]. The miRNA cargo of circulating MVs was also explored in the CM murine model. miR146a and miR193b were shown to be differentially abundant in MVs from CM mice (*P. berghei* ANKA-infected mice) when compared either to non-CM mice (*P. yoelii*-infected) or uninfected controls [61]. Although the functional implications of these two miRNAs were not experimentally assessed, an in silico pathway analysis suggested a regulatory role for these targets in the neurological syndrome associated with ECM [61].

## 3. Leishmaniasis

**Figure 2:**
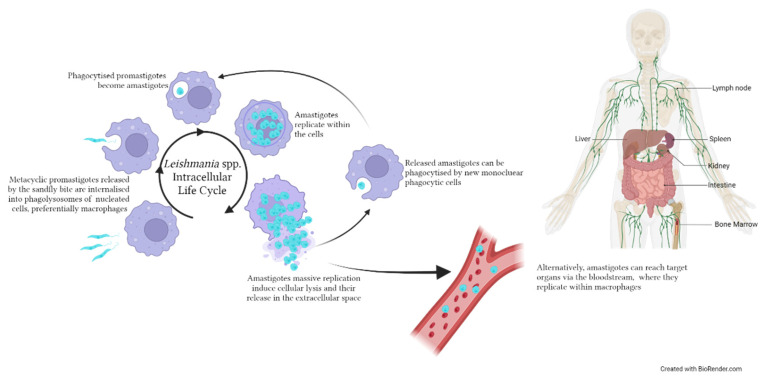
Life cycle of *Leishmania* spp. within the human/canid host. Metacyclic promastigotes are inoculated in the host by an infected sandfly during a blood meal. The intracellular life cycle occurs within nucleated cells, preferentially macrophages, where promastigotes are phagocytised, transformed into amastigotes and start replicating. Following the rupture of the infected cell, amastigotes can invade new mononuclear cells or reach target organs such as the viscera, lymph nodes and bone marrow. The image was created with BioRender.com.

Leishmaniasis is caused by protozoan parasites of the genus *Leishmania*, which comprises more than 20 species pathogenic for humans [78]. From a clinical point of view, leishmaniasis is divided into three main forms: visceral, also called Kala-Azar (lethal if left untreated), cutaneous and mucocutaneous leishmaniasis, both disfiguring and disabling. Leishmaniasis is endemic in 99 countries, and up to 1 million new cases are estimated to occur annually, even though only 200,000 new cases are reported [79]. *Leishmania* spp. are obligate intracellular parasites presenting a digenic lifecycle: a motile flagellate form (promastigote) is present in the gut of the invertebrate vector, and a non-motile non-flagellate form (amastigote) replicates in the infected host (humans or dogs). In the human host, the parasite proliferates within mononuclear phagocytes, and, despite its capability to invade different cell types, it selectively infects alternatively activated macrophages (M2 macrophages), i.e., “effector cells” [80] (Figure 2). The outcome of the infection further depends on the interaction of the parasite with multiple innate immune cells such as neutrophils, natural killer (NK), dendritic cells (DCs) and mast cells, as well as on the adaptive immune response [81]. 

Different mechanisms of host–parasite communication are involved in this interaction, among which EVs released by both *Leishmania* spp. and host cells play an important role [21]. *Leishmania* spp.-derived EVs have been largely studied since 2010, when the ability of this parasite to secrete EVs (or exosome-like vesicles) was first described [82]. Since then, accumulating evidence has supported a functional role of parasite-derived EVs (particularly those released by infected host cells) in parasite survival and in the interaction with and modulation of the host immune response, as reviewed elsewhere [20,83]. Briefly, EVs released by different cell types upon infection with *Leishmania* spp. have been reported to: (i) contain parasite proteins, including virulence factors (e.g., glycoprotein 63 kDa (gp63)) and drug-resistance genes [14,82,84,85,86,87,88]; (ii) protect from or reduce the burden of infection when adoptively transferred in recipient mice [89,90] and (iii) induce changes in gene expression in recipient cells [90,91]. 

### Host-Derived Circulating EVs: The Case of Dogs and Mice

To the best of our knowledge, up to date only a few studies have investigated circulating EVs released by the host during a *Leishmania* infection, focusing mainly on their protein and small RNA cargo (Table 2, Table 3 and Table 4). Dogs infected with *L. infantum* were reported to harbour significantly higher numbers of circulating EVs compared to uninfected controls and were significantly smaller in size [92]. Increased concentrations of miR-21-5p and miR-146a-5p were reported in the serum of the same dogs; unfortunately, only a small selection of miRNAs was assessed, and their abundance within circulating EVs was not investigated. The protein cargo of circulating EVs during canine leishmaniasis was instead investigated by Esteves et al., who compared healthy and *L. infantum*-infected dogs (CanL) [58]. Among the 1148 identified proteins, 46.1% were common to both groups, while 154 proteins (13.41%) were present only in the CanL group. Interestingly, carbonic anhydrase (F1PBK6) and myo-inositol were identified only in healthy dogs (17/19 and 6/19, respectively), while none were identified only in the CanL group. Gene ontology analysis (GO) highlighted two GO terms specifically enriched in CanL-EV, i.e., antigen processing and presentation via MHC class I and calcium ion binding. The former term is particularly interesting since it is consistent with *Leishmania* being an intracellular parasite and with previous observations on the presence of MHC molecules within EVs [9]. It could thus be hypothesised that EVs released in the circulation during leishmaniasis participate in CD8^+^ T cell activation. Twelve *L. infantum* proteins were also identified within plasma-circulating EVs, and putative DNA polymerase epsilon subunit b (LINJ_35_1780) was the most frequently detected (six dogs), even though protein identification was achieved with only one unique peptide [58]. A broader identification of pathogen-derived proteins was likely hampered by the presence of highly abundant plasma proteins. A more extensive removal of plasma protein contamination from the EV preparation could potentially improve the identification of *Leishmania* proteins within circulating EVs; nonetheless, this study provided the first experimental evidence of the presence of *Leishmania*-derived proteins in circulating EVs in vivo. 

The content of small RNAs in circulating EXOs was instead evaluated in mice infected with *L. donovani* or *L. amazonesis* [62]. Using NGS, it was shown that EXOs from infected mice bore rRNAs and tRNAs of parasitic origin. In particular, 71 and 15 small RNA reads were specifically identified in *L. donovani*- and *L. amazonesis-*infected mice, respectively, as they did not match with *Mus musculus*. Even though the study lacked some important characterization of the vesicular population under investigation, to the best of our knowledge, it represents the first evaluation of plasma EV RNA cargo in *Leishmania*-infected mice and the first report on the presence of parasite-derived RNA species within circulating EVs during leishmaniasis. 

Despite some limitations, the studies here presented provide important evidence supporting circulating EVs as a potential source of biomarkers and diagnostic antigens in leishmaniasis. Investigations should now be extended to human clinical samples to better understand the communication at the host-*Leishmania* interface and reveal novel diagnostic markers.

## 4. Chagas Disease

Chagas disease (CD or American trypanosomiasis) is a NTD caused by the protozoan parasite *Trypanosoma cruzi* transmitted to the human host by infected triatomine bugs, even though alternate transmission routes including congenital, transfusion and organ transplantation are well recognised [98,99]. The disease is considered to affect 6–7 million people worldwide, with the highest burden is registered in Latin America [100]. 

The infection usually progresses through a first acute phase, characterised by a high parasite load but asymptomatic in the vast majority of cases (>95%), to a chronic phase, which can last for decades. During this chronic phase (CCD, chronic Chagas disease), 30–40% of patients can, at some point, present symptoms, the most common and indicative of which are cardiomyopathy and megaviscera [98]. Even if the pathophysiological mechanisms of CCD are not fully understood, it has been hypothesised that in asymptomatic CCD patients, the host immune system may play an important role in keeping parasitaemia at low levels. Alterations to this equilibrium might thus lead to an increased parasite load accompanied by excessive immune activation and tissue damage [17].

**Figure 3:**
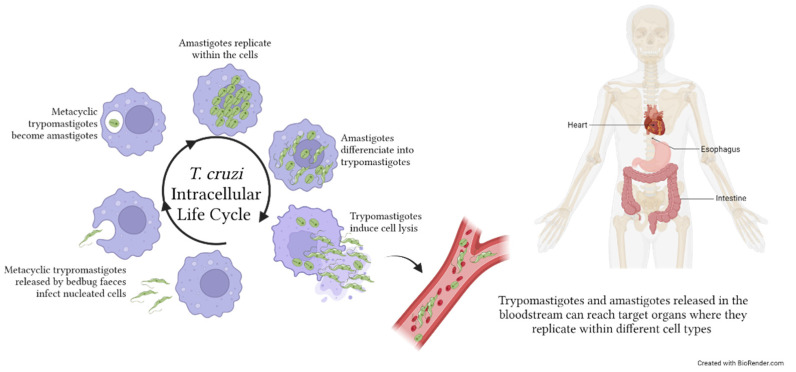
*Trypanosoma cruzi* life cycle within the human host. Metacyclic trypromastigotes are released by bedbug faeces on the host skin. Penetration occurs following scratches on the purulent insect bite wound. Within the host, metacyclic trypomastiogtes infect nucleated cells, become amastigotes, and replicate. The majority of amastigotes become trypomastigotes, inducing the lysis of the infected cells and the consequent release of parasites in the extracellular space. Both trypromastigotes, and amastigotes can reach, through the bloodstream, distant target organs such as the viscera and heart, where they infect tissue cells. *T. cruzi* infection may also affect the skeletal muscles and produce neurological disorders. The image was created with BioRender.com.

As stated in the latest WHO roadmap, a number of issues associated with CD management are still hampering its elimination as a public health problem, including the lack of effective rapid diagnostic tests and biomarkers for the evaluation of treatment outcomes [100]. Within the human host, *T. cruzi* amastigotes multiply intracellularly by binary division and differentiate into trypomatigotes, which will be released in the circulation to infect new cells in a variety of different tissues [101] (Figure 3).

### Circulating EVs in Chagas Disease: Cargo and Functional Properties

In the context of CD, EVs have recently been dealt with in a systematic review of the literature, the objective of which was to describe their contribution to the understanding of disease progression [17]. As for the other diseases described in this review manuscript, we will also focus on circulating host-derived EVs with a special regard for their utility as potential clinical tools or in the pathophysiological context (Table 2, Table 3 and Table 4). 

In 2017, Chowdhury et al. investigated for the first time the potential implication of circulating MVs as mediators of the macrophage (Mφ) response during Chagas disease [93]. Using an in vitro system, plasma from asymptomatic (A-CD) and symptomatic (S-CD) patients, and chagasic mice, they studied the potential of circulating MVs as biomarkers of the host’s inflammatory and oxidative states as well as of disease severity. Increased circulating MVs of monocytic, endothelial and lymphocytic (mainly CD8^+^) origin were reported in both patients and mice during CD infection. Moreover, MVs from CD patients were reported to induce alterations in Mφ gene expression in a disease-stage-dependent manner. Indeed, MVs from symptomatic patients promoted a larger Mφ inflammatory activation compared to asymptomatic patients, as determined by the increased expression of CD14^+^ and CD16^+^ Mφ. MVs from CD patients also induced oxidative and nitrosative stress and the release of soluble cytokines from Mφ, with IL-2 and IFN-γ being predominately induced by symptomatic MVs. These results reinforced the hypothesis of a role for CD-derived MVs as pathophysiological mediators in a stage-dependent fashion. This was further supported by the evidence that MVs from *T. cruzi*-infected mice elicited in Mφ a more pronounced inflammatory response and nitrosative stress compared to MVs from vaccinated mice subsequently challenged with *T. cruzi*. 

With the objective of establishing the potential utility of circulating EVs in the context of treatment-response evaluation, Cortes-Serra and colleagues investigated the protein cargo of plasma-derived EVs in a CD patient who underwent heart transplantation and disease reactivation after immunosuppression [59]. Using tandem mass spectrometry, they showed that patient-derived EVs carried a higher number of proteins compared to healthy controls, and they highlighted four proteins altered in abundance following treatment. Interestingly, pre-treatment CD-EVs harboured one *T. cruzi* protein (identified with high confidence) and 19 human proteins not detected in the other samples. The latter group included mannan-binding lectin serine protease 2 (MASP2), which was already proposed as associated with a higher risk of CD cardiomyopathy [102]. Although those observations were drawn from a single case study, they indicate that circulating EVs might be an important source of disease biomarkers and deserve further analysis.

EV miRNA content was instead investigated using a mouse model, where an increased number of circulating EVs during the acute phase of the infection was observed when compared to uninfected controls [63]. miR21 and miR146a, previously reported to be associated with heart fibrosis and leukocyte infiltration in cardiovascular disorders [103], were reported to be significantly more abundant within EVs from the acute phase [63]. Interestingly, similar profiles were also observed in the cardiac tissue and in whole plasma, even though in these matrixes, alterations in miRNA abundances (miR-21, miR-146a and miR155) were also observed in the indeterminate phase. These data suggested that different mechanisms of cellular activation might occur during the two phases, accompanied by the release of different EVs and with different molecular packing. The over-expression of these miRNAs could represent an important mechanism of gene expression regulation involved in the Mφ inflammatory response; they should thus be further investigated as potential early diagnostic markers. Nonetheless, investigations should also be extended to include untargeted EV-miRNA sequencing.

A few studies have investigated functional aspects of EVs released by host cells in response to infection, suggesting their potential involvement in immune escape and their pro-inflammatory effect. Host-derived MVs were in fact proposed to play an important role in parasite escape from the host-immune response and in the promotion of parasite survival. *T. cruzi* trypomastigotes induced an increased release of MVs in a Ca^2+^-dependent manner from in vitro cultured THP1 and peripheral blood mononuclear cells (PBMCs), and these MVs promoted parasite survival by inhibiting complement-mediated parasite lysis [104]. In agreement with this, EVs derived from blood cells during infection were shown to promote cell invasion, thus allowing parasites to escape from the host immune response. Interestingly, in the murine model, *T. cruzi* infection induced the release of circulating EVs, the exogenous transfer of which promoted the increase in parasitaemia [104]. The ability of infective trypomastigotes to trigger a conspicuous release of MVs from THP-1 monocytes or PBMCs from healthy donors was later confirmed [94]. In particular, MVs released during the interaction of trypomastigotes and THP1 (i.e., the co-culture system) promoted cell invasion in vitro and potentiated early parasitaemia in vivo. The proteomics analysis of the cargo of MVs released during this THP/trypomastigote interaction revealed the presence of both host- and parasite-derived proteins, further supporting the hypothesis that this vesiculation process occurs in concert in the two cell types, the exact timing and kinetics of which have, however, yet to be determined [94]. These MVs were also suggested as a source of immunogenic antigens since they were specifically recognised by antibodies present in CCD patients’ plasma [94]. 

In a recent study, CCD patients were reported to harbour a lower number of circulating EVs compared to healthy controls, as determined by nanoparticle tracking analysis (NTA), but with potential pro-inflammatory properties [95]. Indeed, CCD-derived EVs induced the release of an increased amount of IFN-γ in differentiated THP1 upon in vitro exposure. This suggests that EVs released in the circulation of CCD patients might be involved in the maintenance of disease chronicity via immunomodulation; more in-depth investigations of their functional properties might thus reveal EVs as a novel tool for disease progression monitoring [95]. In contrast, Ramirez et al. using flow cytometry did not observe differences in the frequencies of circulating MVs in CD patients at different phases of disease progression (i.e., chronic indeterminate or cardiac phase) compared to healthy controls [94]. It is actually hard to hypothesise whether these contrasting results are due to methodological aspects or to intrinsic characteristics of the studied population. However, such investigations should also be expanded to include the analysis of specific MV sub-populations. Indeed, surface immunophenotyping analysis of circulating EVs associated with CD has yet to be reported, as has in-depth characterisation of their cargo of proteins and miRNA. 

The preliminary results reported in the literature seem to indicate an interest in studying host-derived EVs in the context of CD. A more comprehensive evaluation of their cargo and surface phenotyping at different stages of infection and in response to treatment might reveal novel biomarkers and novel targets for the development of diagnostic tools, as indicated in the latest WHO recommendations [100].

## 5. Circulating EVs in Human African Trypanosomiasis (HAT): Evidence in CSF

**Figure 4:**
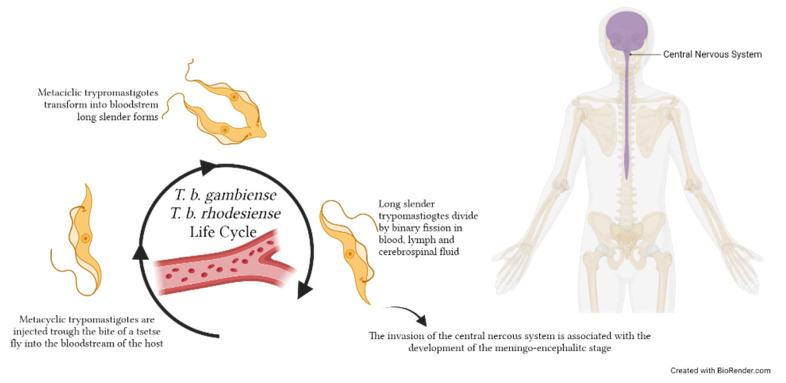
*Trypanosoma brucei gambiense* and *T. b. rhodesiense* life cycles within the human host. Metacyclic trypomastigotes are injected through the bite of a tsetse fly during the blood meal into the host bloodstream, where they replicate extracellularly. Parasites replicate by binary fission as long, slender trypomastigote forms in various body fluids, including blood, lymph and—when they reach the central nervous system—cerebrospinal fluid. The image was created with BioRender.com.

Human African trypanosomiasis, also known as sleeping sickness, is a NTD caused by the extracellular protozoan parasite *Trypanosoma brucei*, which is transmitted through the bite of an infected tsetse fly [105]. Two subspecies, *T. b. gambiense* and *T. b. rhodesiense*, can infect the human host, causing a disease that can be fatal if, when untreated, the parasite reaches the central nervous system [106] (Figure 4). This progression is much faster during an infection caused by *T. b. rhodesiense*. The two parasites are found only in sub-Saharan Africa, and their distribution is geographically separated since *T. b. gambiense* is diffused in Central and West Africa, while *T. b. rhodesiense* is found in East Africa [106]. *T. b. gambiense* is responsible for the majority (up to 92%) of the reported cases. The implementation of important control measures, including active and passive case finding, patients’ treatment and vector control, has led to a drastic reduction of the reported cases during the last decades—especially for *T. b. gambiense*—with less than 1000 cases reported annually in 2019 and 2020 [107], compared to 35,000 cases reported annually at the end of the 20th century [108].

These data indicated that the objective of HAT elimination as a public health problem (i.e., less than 2000 cases per year) was reached [107]; efforts should now be focused on the ambitious goals of *T. b. gambiense* transmission elimination and *T. b. rhodesiense* elimination as a public health problem by 2030, as stated in the latest WHO road map for NTDs [100]. Indeed, being a zoonosis, *T. b. rhodesiense* control requires more complex control measures from a health point of view. 

The pathophysiological mechanisms accompanying disease progression from the hemolymphatic first stage to the meningoencephalitic second stage are yet to be fully understood. The mechanisms driving the passage of parasites through the blood–brain barrier (BBB) are still unclear; however, they seem to involve an initial transient reduction of the BBB integrity followed by a progressive impairment [109]. This neuro-invasion also involves the transmigration of immune cells and the development of a neuro-inflammatory reaction, as demonstrated by the increased pro-inflammatory cytokines and chemokines in the CSF of late-stage patients [109].

To the best of our knowledge, only a few studies have investigated EVs associated with HAT, most of which focused on parasite-derived EVs [110,111,112,113]. Only one study has instead explored host-derived MVs in the cerebrospinal fluid (CSF) of patients with different stages of progression of *T. b. gambiense* HAT [96] (Table 4). Late-stage patients’ CSF was shown to contain a significantly higher number of total MVs as well as leukocyte-derived MVs when compared to stage 1 and intermediate stage (also defined as early late stage). Interestingly, late-stage MVs induced alterations in the protein expression of human astrocytes in vitro similar to those observed in cells exposed to IFN-γ, a pro-inflammatory cytokine involved in S2 HAT [109]. Conversely, cells exposed to early-stage MVs induced a less marked alteration. These results indicate that late-stage HAT is associated with an increased release of MVs in CSF, probably as a result of cell activation and extravasation, and that these MVs may represent novel mediators in the neuro-inflammatory process that accompanies HAT S2.

## 6. Toxoplasmosis

**Figure 5:**
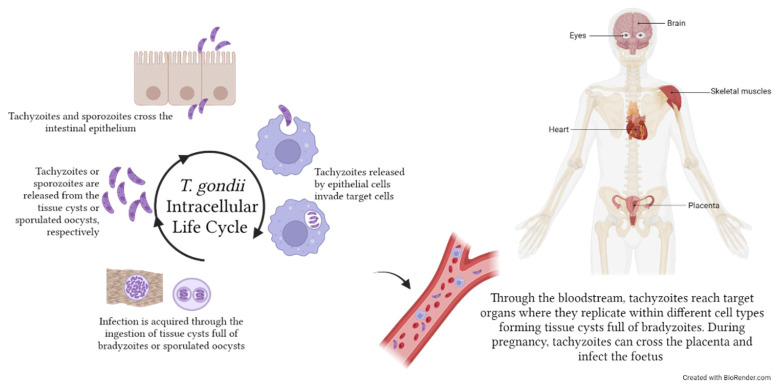
*Toxoplasma gondii* life cycle within the human host. Humans acquire *T. gondii* infection following ingestion of food contaminated with tissue cysts full of bradyzoites or with sporulated oocysts containing sporozoites. Tachyzoites and sporozoites are then released in the stomach and enter epithelial cells, from which they egress as tachyzoites. Tachyzoites can then invade any kind of cell (e.g., monocytes) to replicate by endodyogeny and disseminate to target organs, such as skeletal muscles, eyes, brain and heart, where they eventually form tissue cysts. During pregnancy, *T. gondii* can pass through the placenta and infect the foetus, causing miscarriages or developmental defects. The image was created with BioRender.com.

Toxoplasmosis is a zoonotic disease due to the intracellular apicomplexan parasite *Toxoplasma gondii*. Humans—as any warm-blooded animal—represent intermediate hosts, while felids are the only definitive hosts [114]. The human infection is acquired upon ingestion of oocysts or intracellular cysts present in contaminated soil/water or food, respectively [114]. *T. gondii* replicates within different mammalian cell types by endodyogeny [115]; in the human host, it forms cysts that can persist lifelong (Figure 5). Human toxoplasmosis is widespread worldwide, and two billion people are considered to be chronically infected globally [116,117], even though different strains of *T. gondii* may vary in virulence and geographical distribution [118]. In immunocompetent individuals, the infection is mostly asymptomatic or presents only weak symptoms during the first few weeks of infection. Serious and potentially life-threatening forms can, however, develop, namely congenital and cerebral toxoplasmosis. Congenital toxoplasmosis occurs when the first infection is acquired during pregnancy since the parasite can cross the placental barrier to infect the foetus, causing damages spanning from developmental impairment to miscarriage and stillbirth [119]. In immunosuppressed patients, particularly those with untreated HIV, a reactivation of a latent infection can cause brain lesions and encephalitis [120]. 

As for other intracellular parasites, it is now evident that the release of parasite material within EVs from infected cells represents an important mechanism of host–pathogen interaction. Anyway, it has yet to be fully understood whether this mechanism is important for the parasite to modulate the host immune response or for the host to improve infection control via the exposure of PAMPs to intracellular pathogens. This aspect has also been investigated in several studies in *T. gondii*, where it has been shown that EVs isolated from infected cells (but not from uninfected ones) are able to stimulate an inflammatory response. Since EVs released by different *T. gondii*-infected cells have recently been reviewed, they will not be described in this manuscript [121].

### Circulating EVs in Toxoplasmosis: Evidence in Serum and CSF

Apart from EVs released by *T. gondii*-infected cells, to the best of our knowledge, only one study has so far investigated circulating host-derived EVs in the context of human toxoplasmosis (Table 4). Da Cruz and colleagues made a preliminary evaluation of the concentration of circulating EXOs in serum and CSF from infected (gestational toxoplasmosis and cerebral toxoplasmosis with concomitant HIV infection) and uninfected subjects. In particular, the assessment by NTA of EXOs enriched by ultracentrifugation revealed a significant increase in the number of circulating EVs in serum from infected patients (both cerebral and gestational toxoplasmosis), but not in the CSF [64]. Using a targeted approach, they also reported alterations in the abundance of some specific miRNAs within EXOs isolated from the different clinical conditions. In particular, they reported an increased abundance of miR125b-5p and miR146a-5p in serum-EVs from patients with cerebral and gestational toxoplasmosis when compared with negative serum-EVs, while, compared to toxoplasma-negative/HIV-positive subjects, EVs from patients with cerebral toxoplasmosis and HIV harboured an increased amount of miR155-5p and miR21-5p [64]. This pilot study, although preliminary, suggests that severe toxoplasmosis might be accompanied by an increased release of circulating EVs and an alteration in their cargo. This was also confirmed in the mouse model [97]. Indeed, compared to uninfected control mice, increased amounts of serum EVs were observed in mice following infection with either the RH strain, causing acute disease, or ME-49, a less virulent strain triggering chronic toxoplasmosis. A similar increase in circulating EVs was also reported in mice immunised with *T. gondii* EVs released in vitro. Circulating EVs from toxoplasmosis mice (acute and chronic) were significantly larger in diameter compared to controls. Interestingly, following infection with the ME-49 strain, circulating EVs peaked 15 days post-infection (i.e., during the acute phase) and 60 days post-infection (i.e., during the chronic phase). Further studies should now be carried out using ‘omics approaches for the untargeted characterisation of their molecular cargo as well as for EV comparison during latent/reactivated disease.

## 7. Concluding Remarks and Future Challenges

In this review manuscript, we have summarised the current state of the art regarding the investigation of circulating host-derived extracellular vesicles in blood and tissue protozoan infections. Protozoan parasites are in fact known to communicate with the host through complex mechanisms of host–parasite interaction [16,19,122], which are, however, still largely unknown. As a result of a strategy for immune escape and/or parasite control, host immune cells are the primary players in these interactions. Extracellular vesicles, of both host and parasite origin, are well recognised as important mediators of this communication. In this context, EVs released into the circulation of the infected host can become attractive for multiple reasons. First, their in-depth characterisation (e.g., cargo, surface phenotype, functional properties) can contribute to better understanding how the host responds to the infection, particularly at different stages of disease progression, thus shedding new light on the pathophysiological mechanisms of disease. Second, the characterisation of their cargo and surface phenotype can reveal novel candidate markers for the development of novel diagnostic tests, broadening their utility as liquid biopsies [123]. Indeed, the presence of specific pathogen-derived antigens or the selective packing of host proteins within EVs could be exploited to improve current diagnostic tests. Third, EVs could represent a valuable strategy for vaccine development since host-EVs loaded with parasitic antigens can elicit an important immune response and promote protection against the infection [113,124,125]. 

Despite these important premises, the investigation of circulating host-derived EVs, apart from malaria, seems to be still in its infancy for most protozoan diseases. Indeed, important technical challenges are still associated with EV research, spanning from the enrichment methods to the tools to ascertain their homogeneity, even though the international community is making important efforts to try to standardise and, to some extent, regulate these aspects [7,8]. 

Another important element to consider is the limited availability of clinical samples since most protozoan diseases affect poor communities living in rural areas with limited access to health care systems. This is also accompanied by the limited access to the technology needed for EVs studies (e.g., flow cytometry, transmission electron microscopy, nanoparticle tracking analysis, high-throughput ‘omics platforms) in low and middle-income countries (LMIC) where these diseases are endemic. Finally, the evaluation of EVs functional properties is also particularly challenging since they are strictly linked to EV cargo/composition, which in turn strictly depends on the conditions under which EVs are released. Thus, especially for in vitro experiments, fine changes in the experimental conditions might lead to different EV behaviour. It is then difficult to evaluate whether EVs biological effects are specifically associated with the disease’s pathological state or with the experimental setting. Rigour in experimental design and reporting is thus compulsory in order to make experiments reproducible and the collected evidence reliable. 

Numerous open questions should be addressed in the future, including (i) the mechanisms of selective packing of EV cargo, especially for host-derived EVs carrying pathogen antigens and (ii) the mechanism through which these EVs “select” their target cells. 

As mentioned above, host-derived EVs are being largely studied in malaria with important findings; we believe that this should serve as an example to drive EV research in the context of other parasitic diseases, which are still affecting millions of people worldwide, but particularly in LMIC countries.

## Figures and Tables

**Table 1:** Summary of the studies investigating circulating EV surface phenotypes or functional properties in malaria.

Study Population	Infecting Pathogen	EV Source	Technique for EVs Analysis	Main Observations of the Study	Ref.
Children with malaria (Malawi)	*P. falciparum*	Plasma	FC: CD51^+^ E-MV	▪ Increased E-MVs on admission in children with coma ▪ Decreased E-MVs at discharge vs. admission in CM patients	[38]
Children with malaria (Cameroon)	*P. falciparum*	Plasma	FC: AnV^+^ total MV CD51^+^ and CD105^+^ E-MV CD41^+^ P-MV CD235a^+^ R-MV CD11b^+^ M-MV CD3^+^ Ly-MV	▪ Increased total MVs, P-MVs, R-MVs, M-MVs and L-MVs in CM patients on admission vs. non-CM patients ▪ Decreased total, P-MVs, E-MVs and R-MVs at discharge vs. admission in CM patients ▪ Inverse correlation between P-MVs and coma depth/duration in comatose malaria patients	[39]
Adults with malaria (Thailand)	*P. falciparum* *P. vivax* *P. malariae*	Plasma	FC: AnV^+^ total MV CD235a^+^ R-MV	▪ Increased R-MVs in malaria patients on admission ▪ Increased R-MVs in *P. falciparum* SM vs. healthy controls	[40]
Adults and children with malaria (Ghana)	*P. falciparum*	Plasma	High-speed centrifugation NTA TEM WB FC: AnV^+^ total MV	▪ Increased plasma MVs in uncomplicated malaria vs. healthy controls ▪ Age-dependent relation between circulating MVs and parasitaemia in malaria subjects	[41]
Adults (travellers) with malaria	*P. falciparum*	Plasma	EVs microarray targeting 40 antigens	▪ Altered concentration of CD106^+^, CD81^+^, HLA-DR^+^ and OPN^+^ MVs in malaria patients vs. controls (AUC 0.92–0.97)	[42]
Patients with malaria	*P. vivax*	Plasma	High-speed centrifugation FC: AnV^+^ total MV CD144^+^ E-MV CD41a^+^ P-MV CD235a^+^ R-MV CD14^+^ M-MV CD45^+^ L-MV	▪ Increased number of P-MVs, R-MVs and L-MVs in uncomplicated *P. vivax* patients vs. controls ▪ In *P. vivax* patients the number of circulating P-MVs correlates with clinical manifestations	[43]
Patients with malaria (Brazil and Colombia)	*P. vivax*	Plasma	SEC NTA Bead-based FC Proteomics	▪ Plasma EVs from *P. vivax* patients: (i) are taken up by the spleen and the liver in immunocompetent mice and by human fibroblasts in vitro; (ii) modulate gene expression in human fibroblast and (iii) mediate adhesion of reticulocytes from infected patients	[44]
C57BL/6J mice	*P. berghei* ANKA	Plasma	FC: Anti-platelet antibody	▪ Increased frequency of P-EVs in infected mice vs. uninfected controls ▪ Inverse relation between circulating P-EVs and platelet count	[45]
CBA mice	*P. berghei* ANKA *P. berghei* K173	Plasma	FC: AnV^+^ total MV CD105^+^ E-MV CD41^+^ P-MV CD235a^+^ R-MV CD11b^+^ M-MV CD45^+^ L-MV	▪ The systemic concentration of total MVs peaks at the time of CM onset ▪ Increased E-MVs, P-MVs, R-MVs and M-MVs in ECM vs. non-CM-infected mice ▪ MVs from CM mice sequester in the brain microvasculature of infected recipient mice	[46]

AnV: annexin V; E-MVs: endothelial MVs; P-MVs: platelet MVs; R-MV: red blood cell; MVs; M-MVs: monocyte MVs; Ly-MVs: lymphocyte MVs; L-MVs: leukocyte MVs; OPN: osteopontin; FC: flow cytometry; NTA: nanoparticle tracking analysis; TEM: transmission electron microscopy; WB: Western blot; SEC: size exclusion chromatography; AUC: area under the ROC curve; CM: cerebral malaria; ECM: experimental cerebral malaria; SM: severe malaria.

**Table 2:** Summary of the studies investigating EV protein cargo in blood and tissue protozoan infections.

Study Population	Infecting Pathogen	EV Source	Technique for EVs Analysis	# ID, Host-Derived	# ID, Parasite- Derived	Main Observations of the Study	Ref.
Adults and children with malaria (Ghana)	*P. falciparum*	Plasma	High-speed centrifugation FC: AnV^+^ total MV LC-MS/MS	Patients: 1729 Controls: 234 Culture SN: 333	29, *P. falciparum*	▪ Malaria-MVs contain a significantly higher number of proteins vs. MVs from *P. falciparum* in vitro culture or healthy subjects ▪ 1311 proteins are identified only in EVs from malaria patients	[53]
DBA/1 and C57BL/6 mice	*P. berghei* ANKA	Plasma	High-speed centrifugation FC SEM LC-MS/MS WB	368	2, *P. berghei*	▪ 60 MV proteins are modulated in abundance during ECM ▪ 21 proteins are identified only in ECM-MVs ▪ Increased CA-I and S100A8 in CM MVs in different mouse strains and in a small number of paediatric clinical samples	[54]
BALB/c mice	*P. yoelii* lethal and non-lethal strains	Plasma	Differential UC TEM Bead-based FC LC-MS/MS	259	31, *P. yoelii*	▪ Circulating EXOs from infected mice: (i) are mainly of reticulocyte origin; (ii) contain *P. yoelii* proteins and (iii) elicit the production of parasite-specific antibodies in recipient mice	[55]
FRG huEP chimeric mice	*P. vivax*	Plasma	SEC Bead-based FC (CD5L^+^) NTA Cryo-EM LC-MS/MS	290, *H. sapiens* 234, *M. musculus*	17, *P. vivax*	▪ Circulating EXOs carry liver biomarkers ▪ 23 human proteins are modulated in EXOs from infected mice	[56]
Malaria patients (Brazil and Colombia)	*P. vivax*	Plasma	SEC NTA Bead-based FC LC-MS/MS	533	20, *P. vivax*	▪ Plasma EVs from *P. vivax* patients contain: (i) a lower number of proteins compared to control-EVs and (ii) parasite proteins	[44]
Malaria patients (Colombia and travellers)	*P. vivax*	Plasma	UC + direct immunoaffinity capture (CD71^+^) WB LC-MS/MS Bead-based Luminex assay	440	48, *P. vivax*	▪ *P. vivax* patients harbour systemic reticulocyte-derived EVs ▪ CD71^+^-EVs from *P. vivax* patients contain: (i) proteins involved in malaria pathogenesis; (ii) parasite-proteins and (iii) immunogenic proteins ▪ 73 proteins are modulated in abundance in *P. vivax-*EVs ▪ 17 proteins are detected only in *P. vivax*-EVs	[57]
Dogs	*L. infantum*	Plasma	SEC Bead-based FC NTA (CD5L, CD71 and CD9) TEM LC-MS/MS	1148	12, *L. infantum*	▪ Qualitative and quantitative differences in the protein cargo of EVs from infected dogs vs. healthy dogs ▪ Presence of *Leishmania* proteins within EVs from infected dogs ▪ 154 proteins identified only in infected dog EVs	[58]
Case report: CCD adult patient	*T. cruzi*	Plasma	SEC NTA Bead-based FC LC-MS/MS	338	12, *T. cruzi* (1 with high confidence)	▪ Circulating EVs from one CCD patient vs. healthy controls: (i) contain a higher number of proteins; (ii) contain proteins altered in abundance and (iii) contain parasite proteins ▪ 4 proteins upregulated in pre-treatment EVs compared to post-treatment ▪ 19 proteins identified only in pre-treatment EVs	[59]

#: number; AnV: annexin V; CA-I: carbonic anhydrase I; FC: flow cytometry; NTA: nanoparticle tracking analysis; TEM: transmission electron microscopy; WB: Western blot; SEM: scanning electron microscopy; SEC: size exclusion chromatography; UC: ultracentrifugation; LC-MS/MS: liquid chromatography tandem mass spectrometry; CM: cerebral malaria; ECM: experimental cerebral malaria; CCD: chronic Chagas disease.

**Table 3:** Summary of the studies investigating EVs small RNA cargo in blood and tissue protozoan infections.

Study Population	Infecting Pathogen	EV Source	Technique for EVs Analysis	# Match, Host-Derived	# Match, Parasite-Derived	Main Observations of the Study	Ref.
Malaria patients (Thailand)	*P. falciparum* *P. vivax*	Plasma	UC RT-qPCR	5	na	▪ miR150-5p and miR51b-5p ↑ in *P. vivax*-EVs vs. controls ▪ let7a-5p ↑ in *P. vivax-* and in *P. falciparum-*EVs vs. controls	[60]
CBA mice	*P. berghei* ANKA *P. yoelii*	Plasma	High-speed centrifugation miRNA OpenArray RT-qPCR	431 quantified	na	▪ 23 miRNAs only in ECM-MVs ▪ 60 miRNAs only in control MVs ▪ 15 miRNAs differentially abundant in ECM-MVs vs. non-CM mice or controls ▪ miR146 ↑ in ECM-MVs ▪ miR193b ↓ in ECM-MVs	[61]
BALB/c mice	*L. donovani* *L. amazonensis*	Plasma	Total EXOs isolation kit Small RNA seq	12,268,998 reads in *L. donovani-*mice 11,410,173 reads in *L. amazonensis-*mice	*L. donovani* hits: 126 in *L. donovani-*mice 119 in *L. amazonensis-*mice	▪ Circulating EVs from infected mice carry rRNA and tRNA of parasitic origin ▪ 71 *L. donovani* specific reads in *L. donovani*-mice ▪ 15 *L. donovani* specific reads in *L. amazonensis*-mice	[62]
CD1 mice	*T. cruzi*	Plasma	UC NTA RT-qPCR	3	na	▪ ↑ number of EVs in CD acute phase ▪ ↑ miR21 and miR146a in CD acute phase EVs	[63]
Patients with toxoplasmosis (gestational and cerebral + HIV)	*T. gondii*	Serum CSF	UC NTA TEM WB RT-qPCR	5	na	▪ ↑ circulating serum EVs in toxoplasma-infected patients but not in CSF ▪ miR125b-5p and miR146a-5p ↑ in serum EVs from severe toxoplasmosis ▪ miR155-5p and miR21-5p ↑ in serum EVs from cerebral toxoplasmosis + HIV co-infection	[64]

#: number; na: not applicable; UC: ultracentrifugation; RT-qPCR: reverse transcription quantitative polymerase chain reaction; NTA: nanoparticle tracking analysis; TEM: transmission electron microscopy; WB: Western blot; ECM: experimental murine cerebral malaria; CD: Chagas disease; CSF: cerebrospinal fluid; ↑: increased; ↓: decreased.

**Table 4:** Summary of the studies investigating circulating EV surface phenotypes or functional properties in leishmaniasis, Chagas disease, human African trypanosomiasis and toxoplasmosis.

Study Population	Pathogen	EV Source	Technique for EVs Analysis	Main Observations of the Study	Ref.
Dogs	*L. infantum*	Serum	UC TEM NTA WB	▪ Increased concentration of circulating EVs in infected dogs ▪ Smaller mean size of circulating EVs in infected dogs	[92]
Symtomatic and asymptomatic CD patients C57BL/6 mice	*T. cruzi*	Plasma	High-speed centrifugation FC (AnV, CD14, CD61, CD62E, troponin, CD4 CD8)	▪ Increased circulating M-EVs, E-MVs and CD8^+^L-MVs in CD patients ▪ Evs from CD patients induce alterations in Mϕ in a disease-stage-dependent manner regarding: (i) gene expression, (ii) surface phenotype; (iii) cytokine release and (iv) oxidative stress and nitrate levels ▪ EVs from immunized/infected mice induce a less prominent inflammatory response in Mϕ compared to EVs from infected mice	[93]
CCD patients	*T. cruzi*	Plasma	FC	▪ Circulating EVs do not differ in frequency in patients with CD at different phases of disease progression	[94]
CCD patients	*T. cruzi*	Plasma	UC NTA	▪ CCD patients display a lower number of circulating EVs ▪ CCD patients-EVs display pro-inflammatory properties in vitro	[95]
HAT patients	*T. b. gambiense*	CSF	FC (AnV, CTV, CD105, NSE and CD45) High-speed centrifugation TEM	▪ HAT late-stage patients have increased number of total and leukocyte-derived MVs in CSF ▪ Late-stage MVs induce alterations in the protein expression in human astrocytes in vitro	[96]
A/Sn inbred mice	*T. gondii*	Serum	UC NTA TEM WB	▪ Increased circulating EVs in infected and EV-immunised mice compared to controls ▪ Larger EVs are released in the circulation following infection ▪ Circulating EVs peak 15 and 60 days post-infection	[97]

FC: flow cytometry; NTA: nanoparticle tracking analysis; TEM: transmission electron microscopy; WB: Western blot; UC: ultracentrifugation; CCD: chronic Chagas disease; HAT: human African trypanosomiasis; CSF: cerebrospinal fluid; AnV: annexin V; CTV: cell trace violet.

## Data Availability

Data sharing are not applicable.
